# Supplementary material for: Causal Relationship between Aging and Anorexia Nervosa: A White-Matter-Microstructure-Mediated Mendelian Randomization Analysis
Source: Biomedicines. 2024 Aug 16;12(8):1874. doi: 10.3390/biomedicines12081874 (PMC11351342; doi:10.3390/biomedicines12081874)
Supplement: Supplementary file 1 [file biomedicines-12-01874-s001.zip › Supplementary Figure/Figure S4/Singe SNP plot of MR analysis for TL to ubm-b-1654.pdf]

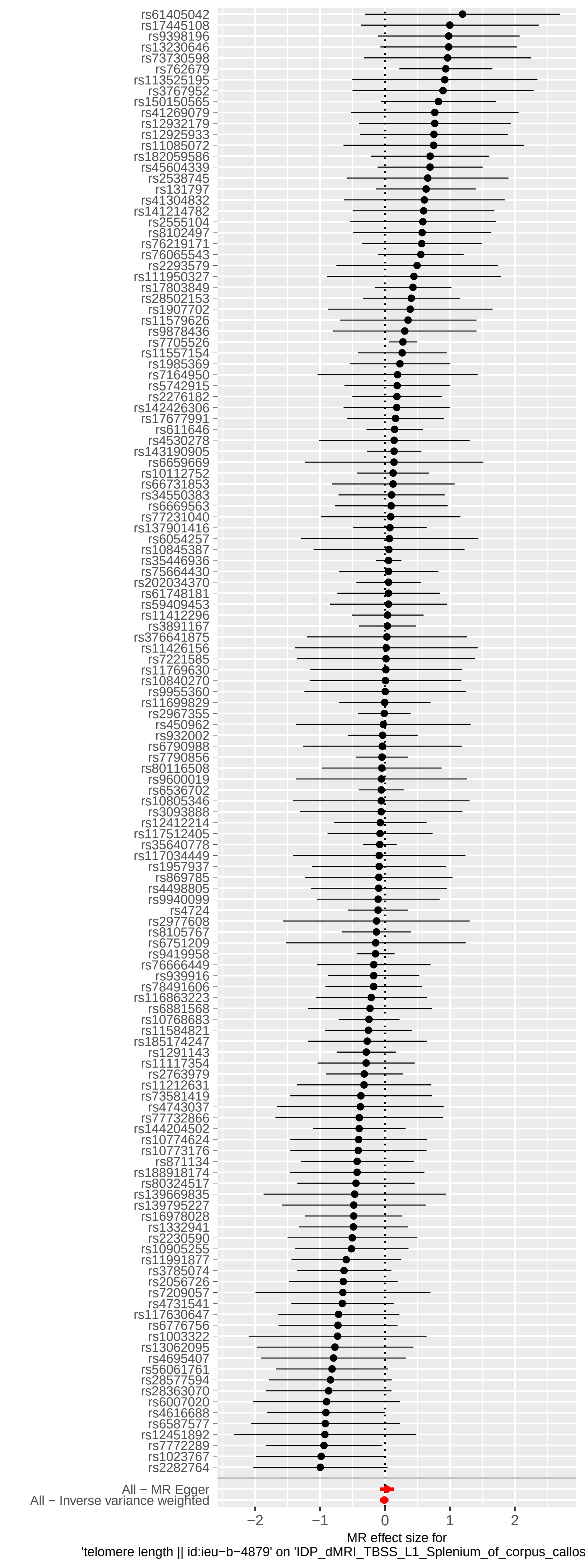
rs61405042
rs17445108
rs9398196
rs13230646
rs73730598
rs762679
rs113525195
rs3767952
rs150150565
rs41269079
rs12932179
rs12925933
rs11085072
rs182059586
rs45604339
rs2538745
rs131797
rs41304832
rs141214782
rs2555104
rs8102497
rs76219171
rs76065543
rs2293579
rs111950327
rs17803849
rs28502153
rs1907702
rs11579626
rs9878436
rs7705526
rs11557154
rs1985369
rs7164950
rs5742915
rs2276182
rs142426306
rs17677991
rs611646
rs4530278
rs143190905
rs6659669
rs10112752
rs66731853
rs34550383
rs6669563
rs77231040
rs137901416
rs6054257
rs10845387
rs35446936
rs75664430
rs202034370
rs61748181
rs59409453
rs11412296
rs3891167
rs376641875
rs11426156
rs7221585
rs11769630
rs10840270
rs9955360
rs11699829
rs2967355
rs450962
rs932002
rs6790988
rs7790856
rs80116508
rs9600019
rs6536702
rs10805346
rs3093888
rs12412214
rs117512405
rs35640778
rs117034449
rs1957937
rs869785
rs4498805
rs9940099
rs4724
rs2977608
rs8105767
rs6751209
rs9419958
rs76666449
rs939916
rs78491606
rs116863223
rs6881568
rs10768683
rs11584821
rs185174247
rs1291143
rs11117354
rs2763979
rs11212631
rs73581419
rs4743037
rs77732866
rs144204502
rs10774624
rs10773176
rs871134
rs188918174
rs80324517
rs139669835
rs139795227
rs16978028
rs1332941
rs2230590
rs10905255
rs11991877
rs3785074
rs2056726
rs7209057
rs4731541
rs117630647
rs6776756
rs1003322
rs13062095
rs4695407
rs56061761
rs28577594
rs28363070
rs6007020
rs4616688
rs6587577
rs12451892
rs7772289
rs1023767
rs2282764
All − MR Egger
All − Inverse variance weighted
−2
−1
0
1
2
MR effect size for
'telomere length || id:ieu−b−4879' on 'IDP_dMRI_TBSS_L1_Splenium_of_corpus_callos
